# Cisplatin-induced caspase activation mediates PTEN cleavage in ovarian cancer cells: a potential mechanism of chemoresistance

**DOI:** 10.1186/1471-2407-13-233

**Published:** 2013-05-10

**Authors:** Mohan Singh, Parvesh Chaudhry, Francois Fabi, Eric Asselin

**Affiliations:** 1 Department of Medical Biology, Research group in Molecular Oncology and Endocrinology, Université du Québec à Trois-Rivières, Trois-Rivières, Québec, Canada; 2 Department of Medical Biology, Université du Québec à Trois-Rivières, 3351 boul.Des Forges, Trois-Rivières, Québec, G9A5H7, Canada

**Keywords:** Cisplatin, Caspases, Cancer, Apoptosis, PTEN

## Abstract

**Background:**

The phosphatase and tensin homolog deleted on chromosome 10 (PTEN) tumor suppressor protein is a central negative regulator of the PI3K/AKT signaling cascade and suppresses cell survival as well as cell proliferation. PTEN is found to be either inactivated or mutated in various human malignancies. In the present study, we have investigated the regulation of PTEN during cisplatin induced apoptosis in A2780, A270-CP (cisplatin resistant), OVCAR-3 and SKOV3 ovarian cancer cell lines.

**Methods:**

Cells were treated with 10μM of cisplatin for 24h. Transcript and protein levels were analysed by quantitative reverse transcriptase-polymerase chain reaction (qRT-PCR) and western blotting, respectively. Immunofluorescence microscopy was used to assess the intracellular localization of PTEN. Proteasome inhibitor and various caspases inhibitors were used to find the mechanism of PTEN degradation.

**Results:**

PTEN protein levels were found to be decreased significantly in A2780 cells; however, there was no change in PTEN protein levels in A2780-CP, OVCAR-3 and SKOV3 cells with cisplatin treatment. The decrease in PTEN protein was accompanied with an increase in the levels of AKT phosphorylation (pAKT) in A2780 cells and a decrease of BCL-2. Cisplatin treatment induced the activation/cleavage of caspase-3, -6, -7, -8, -9 in all cell lines tested in this study except the resistant variant A2780-CP cells. In A2780 cells, restoration of PTEN levels was achieved upon pre-treatment with Z-DEVD-FMK (broad range caspases inhibitor) and not with MG132 (proteasome inhibitor) and by overexpression of BCL-2, suggesting that caspases and BCL-2 are involved in the decrease of PTEN protein levels in A2780 cells.

**Conclusion:**

The decrease in pro-apoptotic PTEN protein levels and increase in survival factor pAKT in A2780 ovarian cancer cells suggest that cisplatin treatment could further exacerbate drug resistance in A2780 ovarian cancer cells.

## Background

The tumor suppressor phosphatase and tensin homolog (PTEN) is negative regulator of the PI3K/AKT pathway [1]. Decrease in PTEN levels could lead to increase in phosphorylation and activation of AKT, which further promotes cell survival and proliferation [2]. Phosphatase activity of PTEN is known to be responsible for the regulation of apoptosis, proliferation and cell migration [3,4]. Epigenetic and genetic changes in PTEN are the crucial factors for PTEN activity and PTEN is mostly found to be deleted or mutated in various human cancers [5]. Ovarian cancer is one of the leading gynecologic malignancy. After surgical intervention for ovarian cancer, cisplatin based chemotherapy is the mainstay for treatment. Major challenge to fight ovarian cancer is the development of chemoresistance. In spite of the extensive research in the field of cancer, certain mechanism of chemoresistance remained unresolved.

Chemotherapeutic drugs like cisplatin are known to act by inducing apoptosis. During apoptosis, a structurally related group of cysteine proteases known as caspases mediate protein cleavage [6,7]. Caspases can be classified into two groups, more precisely initiator and effector caspases. Initiator caspases group includes caspase-6, -8, -9, and −10; they are responsible in initiating a proteolytic cascade by activating the pro-caspases to amplify the death signal. The second group, consists of caspase-2, -3, and −7, are known as effector caspases; they are activated by the initiator caspases [8]. A plethora of caspase substrates have been identified till date and the list is expanding fast [9].

Previous studies suggest that PTEN can be regulated at the transcriptional and post-translational levels through multiple molecular pathways [10-12]. Recently, it has been found that microRNAs can also target PTEN, regulate AKT signaling pathway and induce cisplatin chemoresistance in ovarian cancer cells [13]. Treatment with cisplatin activates the caspases cascades in the cells, which further leads to the induction of apoptosis [14-16]. Recent study from our lab determined that cisplatin induced activation of caspase-3 can cleave tumor suppressor Par-4 protein, associated with selective killing of cancer cells, suggesting that activated caspases could target cellular proteins involved in tumor suppression [9]. It has been shown that caspase-3 can cleave PTEN in HEK293 cellular extracts and furthermore demonstrated that C-terminal cleavage by caspase-3 is negatively regulated by phosphorylation of Ser^370^ and/or Ser^385^[10]. Based on these studies, we hypothesize that cisplatin induced caspase activation could target PTEN in ovarian cancer cells. The outcomes of the present study indicate that cisplatin mediated caspases activation leads to the cleavage of PTEN which results in AKT phosphorylation in ovarian cancer cells suggesting that cisplatin based chemotherapy could induce chemoresistance by targeting PTEN in ovarian cancer cells.

## Results

### Cisplatin treatment decreases PTEN protein levels

**Figure 1:**
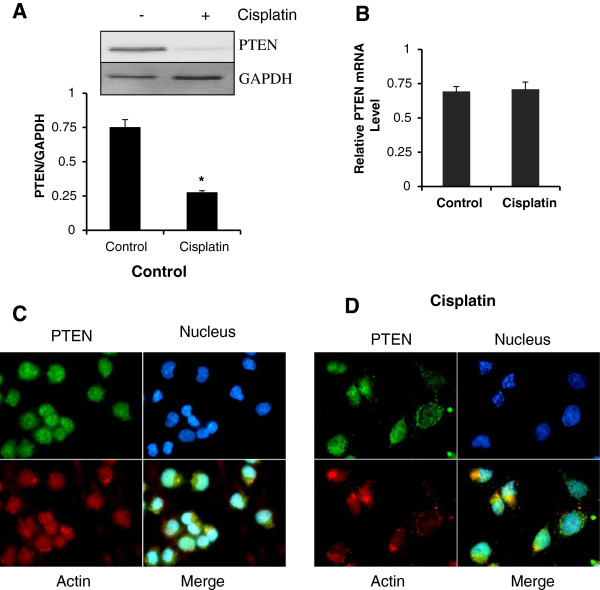
**Post-translational regulation of PTEN by cisplatin treatment.** (**A**) PTEN protein levels in A2780 cells after 24 hrs cisplatin treatment. GAPDH was used as loading control. Graphs show densitometric analysis of three independent experiments. * p < 0.05 compared to vehicle treated cells. (**B**) Real-time quantitative PCR analysis for PTEN relative mRNA levels in A2780 cells after 24 hrs of cisplatin treatment. GAPDH was used as internal control. (**C** &**D**) A2780 cells were grown on glass coverslips and treated with cisplatin for 24hrs. Following treatment, cells were fixed using 4% paraformaldehyde and immunofluorescence was performed using PTEN (Green) antibody. Nuclei were counterstained with Hoechst 33258 (blue) and rodamine phalloidin (red) was used to stain actin cytoskeleton and to visualize the shape and cellular integrity. Magnification: 63 × .

A2780 cells were treated with 10μM cisplatin (based on previous studies from lab) and the results revealed that PTEN protein levels were markedly decreased after 24 h cisplatin treatment (Figure 1A). The time interval for the treatment was based on the time course study (Additional file 1: Figure S1). This decrease in PTEN protein levels could be a result of decreased transcript levels; therefore, we evaluated PTEN mRNA levels. The results of real-time quantitative PCR demonstrated that PTEN transcript levels remain unchanged following cisplatin treatment (Figure 1B). We were further interested to know whether cisplatin treatment also effects the intracellular localization of PTEN (Figure 1C&D). Immunofluorescence analysis confirmed reduced levels of PTEN proteins after cisplatin treatment (Figure 1D). Further, nuclear PTEN levels were found to be decreased in cisplatin treated A2780 cells with membrane localization as seen by yellow color development due to red labeled actin and green labeled PTEN in merged picture (Figure 1D).

**Figure 2:**
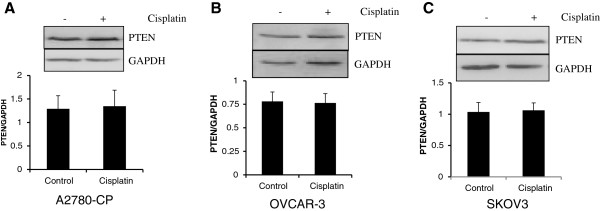
**PTEN protein levels in cisplatin treated resistant and less sensitive ovarian cancer cells.** (**A**) A2780-CP cells (**B**) OVCAR-3 cells (**C**) SKOV3 cells were treated with cisplatin (10μM) for 24 hrs. Protein levels were analyzed using western blotting. GAPDH was used as a loading control. No significant in PTEN protein levels were observed in these cell lines.

Additionally, we also tested various other ovarian cancer cell lines for PTEN levels following cisplatin treatment. The results showed that there was no change in PTEN protein levels in A2780-CP (cisplatin resistant), SKOV3 and OVCAR-3 ovarian cancer cells (Figure 2A-C).

### Cisplatin treatment promotes phosphorylation of AKT

**Figure 3:**
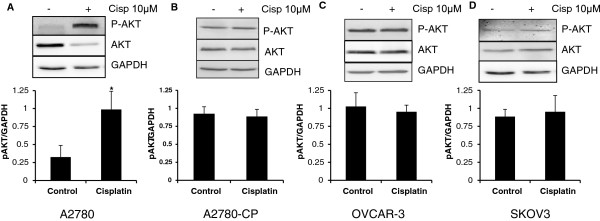
**Cisplatin treatment in A2780 ovarian cancer cells increased phosphorylation of AKT in A2780 cells.** (**A**) A2780 cells (**B**) A2780-CP cells (**C**) OVCAR-3 cells (**D**) SKOV3 cells were treated with cisplatin (10μM) for 24 hrs. pAKT protein levels were analyzed using western blotting. GAPDH was used as a loading control. * p < 0.05 compared to vehicle treated cells.

PTEN is known as a negative regulator of AKT phosphorylation. The phosphorylation of AKT was analyzed using western blotting in various cell lines. Significant levels of phosphorylated form of AKT were observed in case of A2780 cells (Figure 3A). However phosphorylation level of AKT remained unchanged in A2780-CP, OVCAR-3 and SKOV3 cells (Figure 3B-C). This result indicates that in spite of inducing cell death, cisplatin could promote cell survival and proliferation in ovarian cancer cells.

### Proteasomal degradation of PTEN in presence of cisplatin

**Figure 4:**
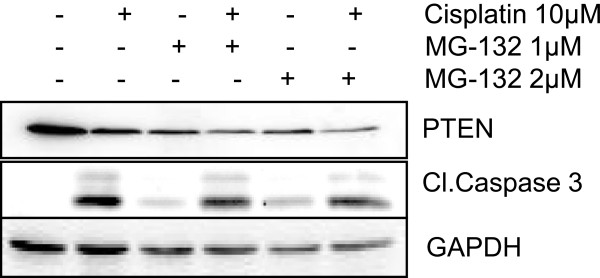
**Cisplatin treatment of ovarian cancer cells does not induce proteasomal degradation of PTEN.** A2780 cells were pre-treated with 1 μM and 2 μM of proteasome inhibitor (MG-132) for 1 hr followed by cisplatin (10μM) treatment for 24 hrs. Protein levels were analyzed using western blotting. GAPDH was used as a loading control.

To ascertain, whether cisplatin mediated decrease of PTEN protein is due to ubiquitin-proteasome pathway, we used MG132, a proteasome inhibitor during present study. A2780 cells were pretreated with MG132 at two different concentrations (1μM & 2μM) for 1h followed by the treatment of cisplatin 10μM for 24h. Immunoblotting revealed that pretreatment with MG132 could not restore PTEN protein levels (Figure 4). These results indicate that cisplatin mediated decrease in PTEN protein levels are not due to induction of proteasomal degradation of PTEN but could be due to some other post-translational mechanism.

### Caspases activation and levels of anti-apoptotic molecules

**Figure 5:**
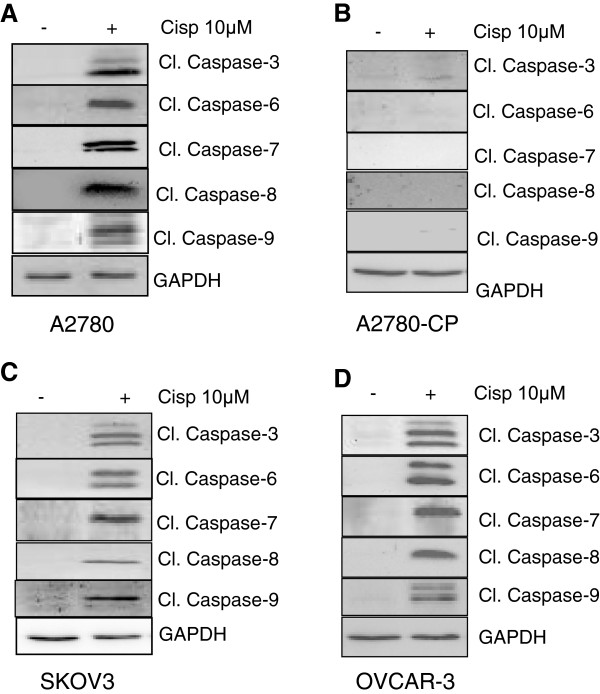
**Cisplatin treatment in ovarian cancer cells activates caspase family members.** Caspases are active in all the cell line tested except cisplatin resistant cell line (A2780-CP). Activated forms of caspases are measured using Western blot assay. GAPDH was used as a loading control.

Caspases are known to be activated during apoptosis induction. In order to understand the differential activation of caspases between the individual cell lines, various caspases were studied. Western blotting results revealed that treatment with cisplatin induced the activation of initiator (caspase-6; caspase-8; caspase-9) and effector caspases (caspase-3; caspase-7) in A2780, SKOV3 and OVCAR-3 cells (Figure 5A, C-D). However, none of the caspases were found to be active in A2780-CP (cisplatin resistant cells) as depicted in Figure 5B.

**Figure 6:**
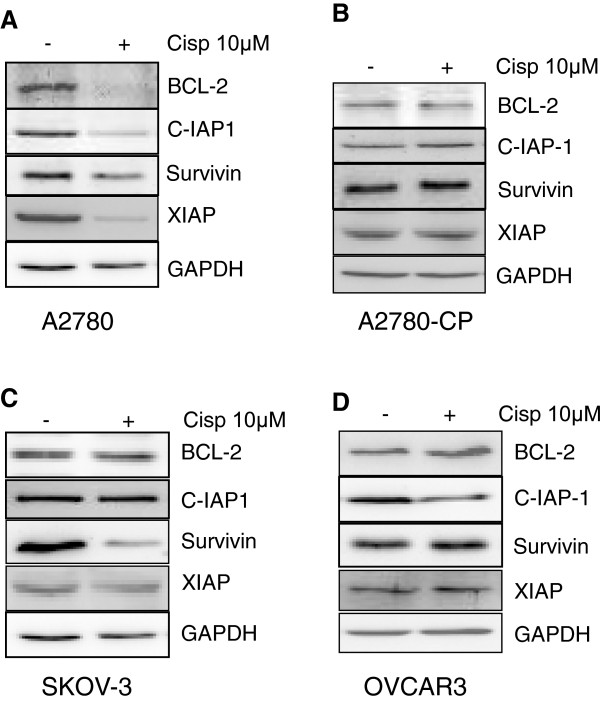
**Levels of Inhibitors of apoptosis (IAPs) in cisplatin treated ovarian cancer cells. (A)** Treatment of cisplatin in A2780 cells decreased BCl-2, XIAP, cIAP1 and survivin protein levels. (**B**) No change in IAPs was observed in A2780-CP cells. (**C**) Decreased levels of survivin in SKOV3 cell lines. (**D**) Decrease in cIAP1 level was observed in case of OVCAR cells. GAPDH was used as loading control.

**Figure 7:**
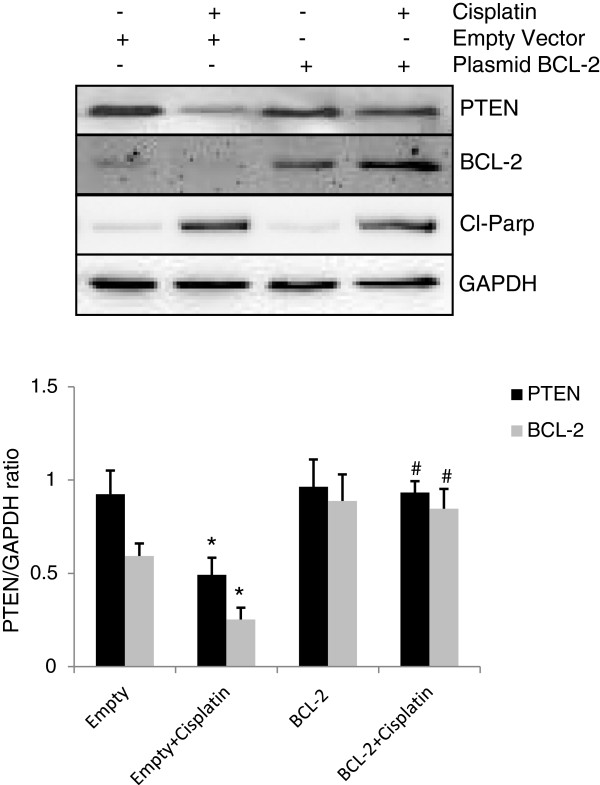
**Overexpression of BCL-2 prevents cisplatin mediated PTEN degradation in A2780 cells.** A2780 cells were transfected with 2ug Bcl-2 plasmid or empty vector. After 48h of transfection, cells were treated with cisplatin and proteins were analyses by western blotting and GAPDH was used as loading control. Graphs show densitometric analysis of PTEN and BCL-2 in various groups. * p < 0.05 compared to vehicle treated cells, # p < 0.05 compared to empty vector transfected cells in the presence of cisplatin.

Inhibitors of apoptosis (IAPs) can directly or indirectly inhibit caspases or pro-caspases [17,18]. For example, XIAP, cIAP1 and cIAP2 can prevent the proteolytic processing of pro-caspases −3, -6 and −7 by blocking the cytochrome c-induced activation of pro-caspase-9. Survivin can bind specifically to the terminal effector cell death proteases, caspase-3 and −7. Furthermore, IAPs can also inhibit caspase-3 directly and thus blocking downstream apoptotic events. We have analyzed the levels of various inhibitors of apoptosis to find out the difference among A2780 and other cell lines tested in this study. Decrease in the protein levels of BCL-2, cIAP-1, survivin and XIAP were observed upon cisplatin treatment in A2780 cells, showing sensitivity towards cisplatin treatment (Figure 6A). On the other hand, no changes were observed in the levels of various IAPs in A2780-CP cells, owing their resistance towards cisplatin treatment (Figure 6B). Among all the IAPs tested, only survivin protein levels in case of SKOV3 (Figure 6C) andcIAP-1 protein levels in case of OVCAR-3 cells were found to be decreased (Figure 6D) suggesting that Bcl-2 protein levels could regulate the caspase activation in A2780 cells. In order to confirm this, we have overexpressed Bcl-2 in the A2780 cells (Figure 7). Interestingly, overexpression of Bcl-2 blocked cisplatin mediated decrease in PTEN protein level. Overexpression of Bcl-2 could resist cisplatin induced apoptosis by blocking the release of cytochrome c from mitochondria thereby inhibiting the activation of downstream caspases which could be involved in the degradation/cleavage of PTEN.

### Role of caspases in PTEN protein degradation

**Figure 8:**
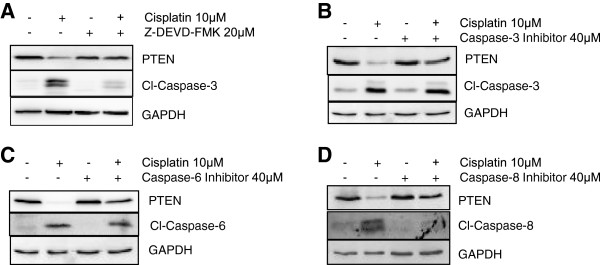
**Pre-treatment with caspase inhibitor restores PTEN levels. (A)** A2780 cells were pre-treated with broad range caspase inhibitor (Z-DEVD-FMK) for 1hr at concentration of 20μM followed by 10μM cisplatin treatment for 24hrs. (**B)** A2780 cells were pre-treated with caspase-3 inhibitor (40 μM) for 1hr followed by 10μM cisplatin treatment for 24hrs. **(C)** A2780 cells were pre-treated with caspase-6 inhibitor (40 μM) for 1hr followed by 10μM cisplatin treatment for 24hrs. **(D)** A2780 cells were pre-treated with caspase-8 inhibitor (40 μM) for 1hr followed by 10μM cisplatin treatment for 24hrs. Protein levels were analyzed using western blot analysis. GAPDH was used as a loading control.

As caspase activation leads to the proteolytic cleavage of various substrates [9] and the results in Figure 5 also demonstrate that various caspases were found to be activated in A2780 cell line, therefore, we determined whether caspase activation could mediate the decrease in PTEN protein levels using caspases inhibitors. Caspases inhibitors act by binding to the active site of caspases either in a reversible or irreversible manner but they do not affect the protein levels of caspases. A2780 cells were pretreated with 20μM of broad range caspase inhibitor (Z-DEVD-FMK) and subsequently treated with 10μM of cisplatin for an additional 24h. Pre-treatment with broad range caspases inhibitor significantly restored PTEN protein levels in cisplatin treated A2780 cells (Figure 8A). This result indicates the potential role of caspases in PTEN degradation upon cisplatin treatment. Furthermore, to determine the involvement of specific caspase in PTEN protein degradation/cleavage, we pretreated the A2780 cells with 40μM of caspase-3 inhibitor for 1h followed by cisplatin treatment (Figure 8B). Pre-treatment with caspase-3 inhibitor restored the PTEN protein levels in A2780 cells. Similar results were observed, when A2780 cells were pretreated with specific inhibitor of caspase-6 and caspase-8 (Figure 8C-D). Collectively, these results suggest that PTEN is a novel substrate of multiple initiator and effector caspases in ovarian cancer cells. Further, PTEN decrease during cisplatin treatment could be the key factor involved in developing chemoresistance in ovarian cancer cells.

## Discussion

PTEN is a putative tumor suppressor protein and a key regulatory molecule of AKT signaling pathway. PTEN possesses lipid phosphatase activity against 3-phosphoinostides opposing PI3K, finally negatively regulating AKT phosphorylation [19]. In the present study, we demonstrate the role of caspases in the regulation of PTEN levels during cisplatin induced apoptosis. In this study we have found that cisplatin induced activation of multiple caspases leads to proteolytic cleavage of PTEN in A2780 cells. Cisplatin treatment induced PTEN degradation in A2780 cells is indicative of post-translational regulation. The activation of AKT by PIP3 production initiates multiple signaling pathways by phosphorylating various downstream targets and by inactivating the inhibitors of cell cycle, protein synthesis glycolysis and angiogenesis. Summarily, it can be said that AKT paves the way for oncogenesis [20,21]. The decrease in PTEN levels leads to the activated form of AKT which could further promotes cellular proliferation and survival in A2780 cells. We have not observed any change in AKT phosphorylation in A2780-CP, OVCAR-3 and SKOV3 cells which could be due the fact that there was no change in the PTEN levels, suggesting that there is a direct relationship between these two proteins in ovarian cancer cells. In addition, cisplatin prevents the nuclear localization of PTEN in A2780 cells which is in accordance with our previous study. In the latter study XIAP knockdown prevents nuclear localization of PTEN, we have also observed that XIAP levels are decreased upon cisplatin treatment which could prevent the nuclear localization of PTEN in the present study. Proteins can undergo proteasomal degradation under external stimuli [22,23]. To validate this hypothesis, we pretreated the cells with MG132, a proteasomal inhibitor and subsequently treated with cisplatin. However there was no restoration of PTEN levels in presence of MG132 and cisplatin (Figure 4; *Lane 4*). Low levels of PTEN was also observed in the only MG132 treated cells because MG132 itself is an apoptotic agent, which further activates caspase-3 (Figure 4; *Lane 3*) and this activation of caspase −3 could lead to a decrease in the level of PTEN as compared to control (*Lane 1*). This result is in accordance with previously published report [12]. Collectively the results from the present study suggest that PTEN does not undergo proteasomal degradation in the presence of cisplatin in A2780 cells.

Cisplatin treatment can initiate both the intrinsic and extrinsic pathways of caspases activation [24]. The activation of various initiator and effector caspases in A2780, OVCAR-3 and SKOV3 cells except A2780-CP cells is indicative of the activation of both apoptotic pathways. However, no particular caspases activation difference was observed among individual cell lines. We could not find out the involvement of any particular caspase in the PTEN degradation from these results. Cell fate is determined by a delicate balance between pro-apoptotic and anti-apoptotic factors [25]. XIAP can inhibit caspase-3 and caspase-7 by directly binding to them [26]. Previous studies have shown that IAPs can inhibit caspases directly or indirectly [17,18] and we have shown that XIAP overexpression can induce chemoresistance in A2780 cells, while XIAP antisense downregulation leaded to increased sensitivity in A2780-CP cells [27]. All the IAPs (BCL-2, cIAP-1, survivin and XIAP) studied in A2780 cells were found be decreased upon cisplatin treatment. However, decreased survivin levels were observed in SKOV3 cells and decreased in cIAP-1 protein levels were seen in OVCAR-3 cells in the presence of cisplatin. As PTEN levels remained stable in SKOV3 and OVCAR-3 cells, we could rule out the role of survivin and c-IAP-1 in caspase mediated PTEN degradation. However, we have observed low endogenous level of BCL-2 in A2780 cells and furthermore BCL-2 level was almost diminished after cisplatin treatment. Decreased levels of BCL-2 could be the reason for higher activation of caspases in A2780 cells owing greater sensitivity than other cell line tested and cleavage of PTEN by activated caspases. Finally, pretreatment with specific caspases inhibitors restored PTEN levels in cisplatin treated cells suggesting the involvement of more than one caspase in PTEN degradation. This result further suggests that PTEN protein sequence contains multiple cleavage sites.

## Conclusions

This study provides the first evidence that PTEN protein can be targeted during cisplatin induced caspases activation in A2780 cells. Caspases-mediated decrease in PTEN levels further affect AKT signaling pathway, which plays an important role in regulating chemosensitivity in ovarian cancer. The present study could provide new insights to understand cisplatin-induced chemoresistance in ovarian cancers and could explain underlying mechanisms involved in PTEN regulation.

## Methods

### Cell culture

Human ovarian cancer cell lines A2780, A2780-CP (cisplatin resistant), cells were cultured in Dulbecco’s modified Eagle’s medium (DMEM/F12) supplemented with 2% BGS (Bovine Growth Serum) (Thrmo Scientific, Rockford, IL) and 50μg/ml of gentamicin. OVCAR-3 cells were cultured in RPMI-1640 supplemented with 10% FBS (Fetal Bovine Serum) (Thrmo Scientific, Rockford, IL) and 50μg/ml of gentamicin. SKOV3 cells were cultured in Mc-Coy’s medium supplemented with 10% FBS and 50μg/ml of gentamicin.

### Reagents and antibodies

AKT total (9272), phospho-AKT (9271), BCL-2 (2872), C-IAP1 (7065), cleaved-caspase-3 (9661), cleaved-caspase-6 (9761), cleaved-caspase-7 (9491), cleaved-caspase-8 (9748), cleaved-caspase-9 (9505), PTEN (9559), phospho-PTEN (9554), Survivin (3879) and XIAP (2042) antibodies were purchased from Cell Signaling (Danvers, MA). Anti-GAPDH(HRP) antibody (9385) was procured from Abcam Inc. (Cambridge, MA) Cisplatin, Proteasomal inhibitor (MG132), and Hoechst 33248 were obtained from Sigma-Aldrich (St. Louis, MO). Broad range Caspase-3 Inhibitor II [Z-DEVD-FMK (264156)], Caspase-3 Inhibitor VII (219012), Caspase-6 Inhibitor I [Z-VEID-FMK (218757)] and Caspase-8 Inhibitor I [IETD (218773)] were obtained from Calbiochem (San Diego, CA).

### Western blot analysis

Following different treatments cells were washed with PBS and submitted to lysis in cold radioimmune precipitation assay (RIPA) lysis buffer containing protease inhibitors (Complete™ from Roche Applied Science) followed by three freeze-thaw cycles. Equal amounts of cell lysates (as determined using Bio-Rad DC protein assay) were separated onto 10%-15% polyacrylamide gels and then transferred onto nitrocellulose membranes (Bio-Rad, Hercules, CA). The membranes were blocked with 5% milk in PBS containing 0.05% Tween 20 for 1h at room temperature, overnight incubated with primary antibody, washed in PBS with 0.05% Tween 20, and probed with horseradish peroxidase-conjugated secondary antibody (Bio-Rad, Hercules, CA). Protein detection was performed using SuperSignal West Femto™ substrate (Thremo Scientific, Rockford, IL), as described by the manufacturer.

### RNA isolation and quantitative-RT-PCR (Reverse Transcription-Polymerase Chain Reaction)

Total RNA was isolated from cells using Purelink™ RNA Mini Kit (Cat no. 12183020 Invitrogen, Carlsbad, CA) according to the manufacturer's instructions. First strand cDNA was synthesized from 1μg of RNA using qScript™ cDNA Supemix (Quanta Biosciences Inc. Gaithersburg, MD). Primers used for amplification were as follows: (i) PTEN forward 5’-ACCCCTTCATTGACCTCAACTA-3’ and reverse 5’-TCTCGCTCCTGGAAGATGGTGA-3’ (ii) GAPDH forward 5’-TGAAGGCGTATACAGGAACAAT-3’ and reverse 5’-CGGTGTCATAATGTCTTTCAGC-3’. PCRs were conducted in LightCycler (Roche). Data were analyzed by using LightCycler Software Version 4.1.

### Transient transfection using BCL-2 plasmid

BCL-2 (pcDNA3 BCL-2) and empty (pcDNA3) plasmids were purchased from Addgene. One day before transfection, cells were plated at 3×10^5^/well to achieve a confluency of ~70% .Next day cells were transfected with 2μg of expression vector using Fugene6 (Roche, Indianapolis, IN) according to manufacturer’s instructions. Cells were incubated for 48h at 37°C, and the medium was replenished with fresh medium containing cisplatin (10μM). The plates were incubated for an additional 24h before the cells were collected.

### Confocal immunofluorescent analysis

Cells were grown on to sterile coverslips in 6 well plates. After cisplatin treatment, cells were fixed with 4% paraformaldehyde for 10min, and washed twice with PBS for 5min. Cells were permeabilized using permeabilizing solution (0.1% Triton, 0.1% sodium citrate) for 10min followed by incubation with Dako blocking serum for 1h. After blocking, cells were incubated with the PTEN primary antibodies or isotypic control antibodies. Both were diluted at a ratio of 1/100 for 1h. After washing with PBS, cells were incubated with fluorescent tag conjugated secondary antibodies (as mentioned in figure legends) for 30min in dark. Cells were counter stained with Hoechst 33248 (0.25μg/ml) for 5min, slides were mounted using slowfade gold anti-fading reagent (Invitrogen) and viewed under Carl Zeiss Axio observerZ1 microscope.

#### Statistical analysis

All the experiments were repeated three times. Data were subjected to one-way ANOVA (PRISM software version 4.0; GraphPad, San Diego, CA) followed by Newman-Keuls test to determine the differences between the experimental groups. Differences were considered significant at the level of *P* < 0.05.

## Competing interests

The authors declare that they have no competing interests.

## Authors’ contributions

MS carried out the experiments, drafted and finalized writing of the manuscript. PC participated in the design of the study, carried out some of the experiments and part of the writing. EA participated in the design of the study and its writing. All the authors have read and approved the final manuscript.

## Pre-publication history

The pre-publication history for this paper can be accessed here:

http://www.biomedcentral.com/1471-2407/13/233/prepub

## Supplementary Material

Additional file 1: Figure S1 Cisplatin treatment decreases PTEN protein levels in a time dependant manner. Cells were treated with cisplatin (10 μM) for increasing time intervals (2, 4, 8, 16 and 24h). Total proteins were then extracted and analysed by Western blot using a PTEN antibody. GAPDH was used as the loading control. Densitometric analysis was performed to quantify protein levels. Click here for file
