# Supplementary figures and images for: Cisplatin-induced caspase activation mediates PTEN cleavage in ovarian cancer cells: a potential mechanism of chemoresistance
Source: BMC Cancer. 2013 May 10;13:233. doi: 10.1186/1471-2407-13-233 (PMC3661380; doi:10.1186/1471-2407-13-233)

## Slide 1
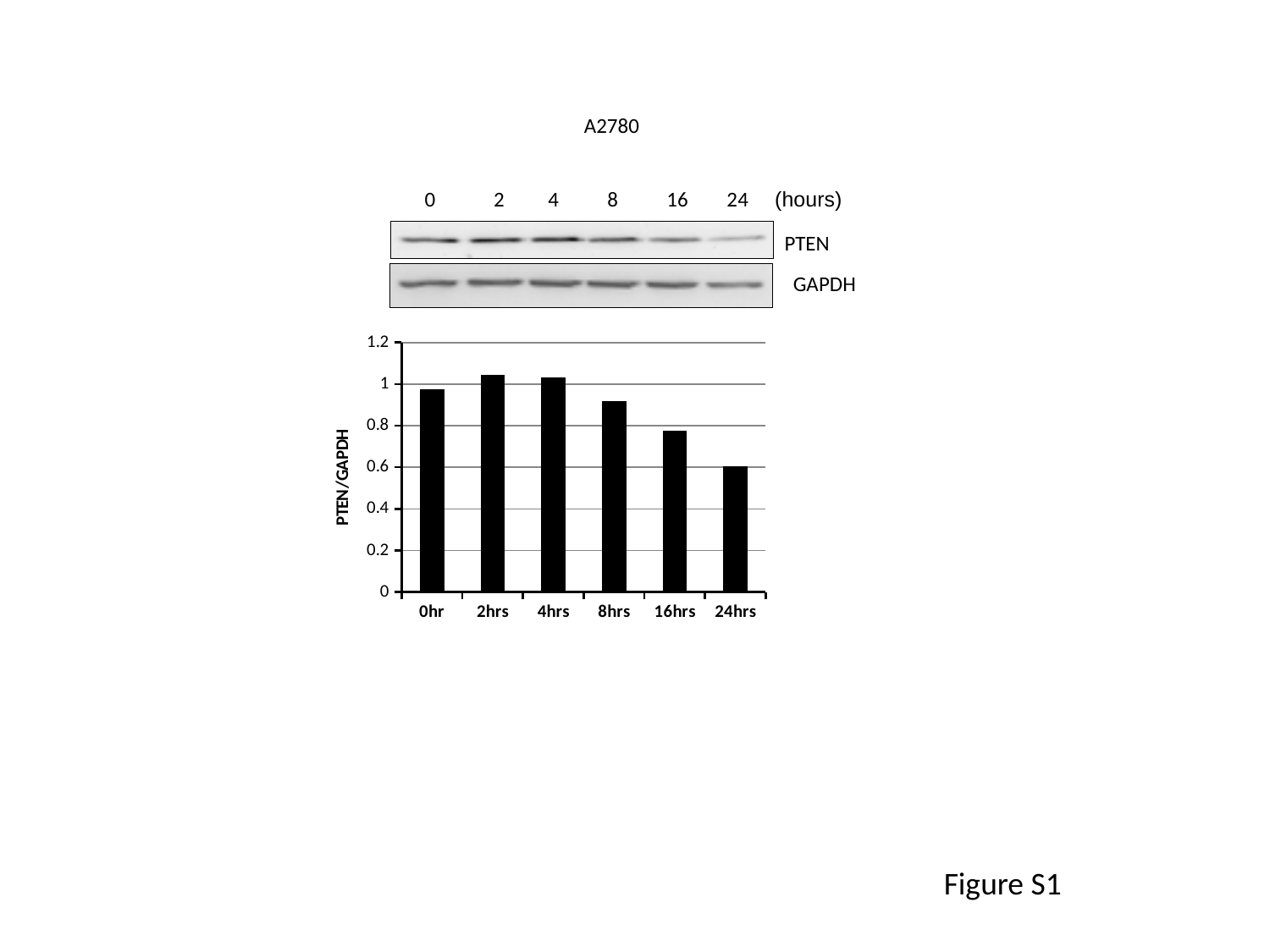

A2780
0 2 4 8 16 24 (hours)
PTEN
GAPDH
### Chart
| Category | |
|---|---|
| 0hr | 0.9770431553964056 |
| 2hrs | 1.044248023824686 |
| 4hrs | 1.0312289571192332 |
| 8hrs | 0.9174518965753261 |
| 16hrs | 0.7762013330049828 |
| 24hrs | 0.6053559295329436 |Figure S1

Supplement: Additional file 1: Figure S1 — Cisplatin treatment decreases PTEN protein levels in a time dependant manner. Cells were treated with cisplatin (10 μM) for increasing time intervals (2, 4, 8, 16 and 24h). Total proteins were then extracted and analysed by Western blot using a PTEN antibody. GAPDH was used as the loading control. Densitometric analysis was performed to quantify protein levels. [file 1471-2407-13-233-S1.pptx]
